# Adverse Drug Reactions in Norway: A Systematic Review

**DOI:** 10.3390/pharmacy7030102

**Published:** 2019-07-25

**Authors:** Mojtaba Vaismoradi, Patricia A. Logan, Sue Jordan, Hege Sletvold

**Affiliations:** 1 Faculty of Nursing and Health Sciences, Nord University, 8049 Bodø, Norway; 2 Faculty of Science, Charles Sturt University, Bathurst 2795, NSW, Australia; 3 College of Human and Health Sciences, Swansea University, Swansea SA2 8PP, UK

**Keywords:** adverse drug reactions, patient safety, nursing, medicines management, healthcare provider, ADR

## Abstract

Prescription medicines aim to relieve patients’ suffering but they can be associated with adverse side effects or adverse drug reactions (ADRs). ADRs are an important cause of hospital admissions and a financial burden on healthcare systems across the globe. There is little integrative and collective knowledge on ADR reporting and monitoring in the Norwegian healthcare system. Accordingly, this systematic review aims to investigate the current trends in ADR reporting, monitoring, and handling in the Norwegian healthcare system and describe related interventions. Appropriate keywords, with regard to ADRs in both English and Norwegian languages, were used to retrieve articles published from 2010 to 2019. Six articles met the inclusion criteria. The findings offer a comprehensive picture of ADR reporting and monitoring in the Norwegian healthcare system. Psychotropic medicines were most commonly implicated by patients, while professionals most commonly reported ADRs associated with anticoagulants. The current ADR systems were compiled with the involvement of both patients and healthcare providers to record all types of drugs and ADRs of various severities, and aimed at improving ADR tracking. However, there is a need to improve current initiatives in terms of feedback and quality, and more studies are needed to explore how ADR profiles, and the associated vigilance, can improve the safety of medicines management in Norway.

## 1. Introduction

The prescription and administration of medicines aims to relieve patients’ suffering and ailments. However, they can be associated with adverse side effects or adverse drug reactions (ADRs) that lead to physical and psychological harm, much of which can be prevented. An ADR is defined by the World Health Organization (WHO) as an unintended reaction in the patient to a medicine associated with any dose administered by the healthcare provider [1]. Serious ADRs are those that can result in life-threatening conditions, persistent or significant disability, prolonged hospitalization, congenital anomalies or death [2]. Efforts have been made world-wide to reduce the number of preventable ADRs [3,4,5]. ADRs cause 5–8% of unplanned hospital admissions in the UK and cost £2.5 billion each year [6,7]. Many patients benefit from prescribed medicines [8], but the high prevalence of ADRs (7.2–8.4%) in community or ambulatory care settings is a challenge to successful medicines management and patient safety strategies [9]. 

ADRs are an ever increasing and substantial burden on healthcare systems and the leading causes of hospital readmissions and associated co-morbidities. Older people [10], patients with multiple comorbidities, polypharmacy, renal impairment, heart failure, and HIV/AIDS [11] are at particular risk. One in eight older patients are re-hospitalized because of an ADR within 12 months of discharge [12]. One in four individuals over 65 years may experience an ADR during hospitalization and one third are classified as severe. However, clinical outcomes associated with ADRs are poorly described [13]. In addition to the direct and indirect costs of care, ADRs reduce patients’ quality of life and wellbeing [14]. The inability to distinguish between ADRs and patients’ underlying diseases can result in further prescriptions, multi-medication, and enhanced risks of drug–drug and drug–disease interactions [15,16]. In European countries, the median percentage of hospital admissions due to an ADR has been reported as 3.5% and the median percentage of patients who experience an ADR during hospitalization is 10.1%. This high prevalence of ADRs in European hospital settings inevitably leads to prolonged hospitalization [17].

The safety of medicines cannot be fully established until they are prescribed to patients and related reactions come under surveillance through well-established pharmacovigilance systems [18]. As a global campaign, the World Health Organization (WHO) 3rd Global Patient Safety Challenge on Medication Safety calls for the development and examination of strategies for addressing concerns regarding medicines’ harms due to medication errors and insufficient monitoring. Since 1971, the international system for monitoring ADRs using information derived from member states has been established under the supervision of the WHO, and the operational responsibility remains with the WHO’s Collaborating Centre for International Drug Monitoring, Uppsala Monitoring Centre, (UMC) in Sweden. Countries with previously established national systems for spontaneous ADR reporting agreed to contribute data based on a common reporting form, guidelines, and classifications. Currently, the ADR database in Uppsala contains several million reports of suspected ADRs [19]. Innovative strategies should be developed to meet current challenges in medication safety [5,20]. For example, a thorough description and reporting of ADRs can help with the development of interventions for the improvement of medicines management [21]. 

### ADR Reporting and Handling in the Norwegian Healthcare System

In Norway, ADRs are estimated to be responsible for 5–10% of all acute internal medicine hospitalizations and cause approximately 1000 deaths per year [22]. Pharmacovigilance and ADR reporting have been defined in Norwegian legislation: pharmaceutical companies, doctors, and dentists are obligated to report on severe or unexpected ADRs [23]. Additionally, other healthcare staff and patients are recommended to spontaneously report ADRs [24]. The Norwegian Medicines Agency (NoMA) has the overall responsibility for pharmacovigilance based on the national ADR reporting system and a database to report ADR data to international databases at the WHO and the European Medicines Agency (EMA) [24]. A national network consisting of four regional medicines information and pharmacovigilance centers (RELIS) handles and assesses ADR reports submitted by healthcare professionals [25]. An annual report is published by NoMA and RELIS: for example in 2018, 5623 ADRs were reported (1.06 ADRs reports per 1000 inhabitants), of which 3% had a fatal outcome [26]. The number of reports increased by 51% from 2017 due to changes in the regulations for reporting ADRs by the pharmaceutical industry, which is now obligated to report all ADRs [26]. However, spontaneous ADR reporting by patients and healthcare staff relies entirely on an individual’s motivation and consequently a significant level of under-reporting remains in the healthcare system [27]. There is little integrative and collective knowledge on ADR reporting and monitoring in the Norwegian healthcare system. Therefore, this systematic review aims to investigate the current trends in ADR reporting, monitoring, and handling in the Norwegian healthcare system and describe related interventions. 

## 2. Materials and Methods 

### 2.1. Design of the Study

A systematic review was undertaken to ensure the comprehensive coverage of the literature and integrate current knowledge [28,29,30] on ADR reporting and monitoring in the Norwegian healthcare system. 

### 2.2. Search Strategy and Data Collection

A pilot search in national and international databases identified appropriate keywords for the search. Next, the Boolean search method was used to retrieve articles describing the condition of ADR reporting and handling in Norway as follows: “Drug-related side effects and adverse reactions” OR “adverse drug event” OR “adverse drug reaction” OR “drug side effects” OR “drug toxicity” OR “side effects of drugs” OR “toxicity, drug” OR “medication side effect” AND Norway OR Norwegian. These keywords were translated to Norwegian and were used for a similar search process in Norwegian research databases. 

Scientific articles published between 2010 and 2019 in journals in both English and Norwegian languages were retrieved from the online databases of PubMed [including Medline], Embase, Cinahl, Web of Science, Cochrane, Norat, Idunn, and SweMed. Inclusion criteria were a precise focus on ADRs in the Norwegian medicines management context and published in scientific peer-reviewed journals. 

### 2.3. Progression of Systematic Review and Quality of Studies

The authors (MV, HS) performed the systematic review steps independently, but held frequent discussions for sharing results of their search and agreed further review steps. This led to retrieval of 6559 articles (Table 1). 

**Figure 1:**
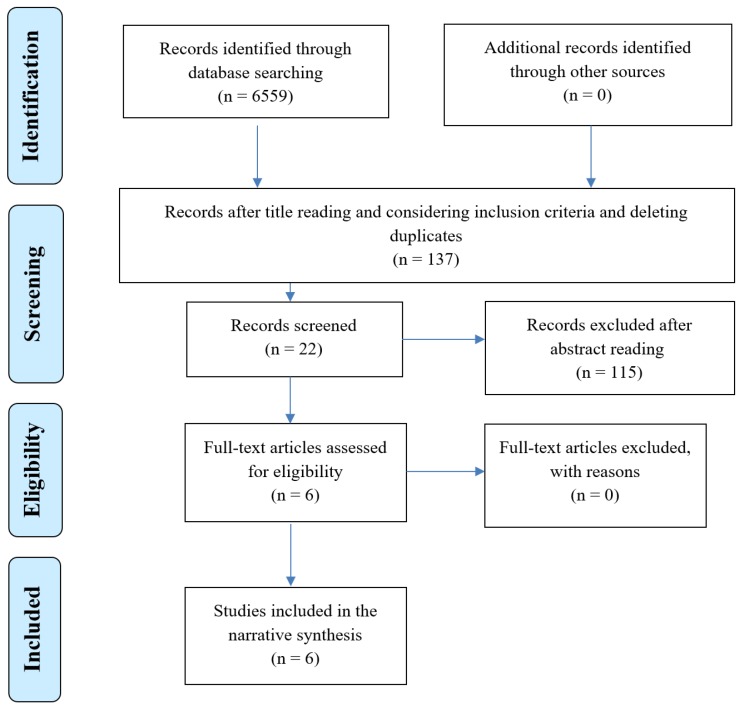
The study selection flow diagram according to the Preferred Reporting Items for Systematic Reviews and Meta-Analyses (PRISMA) flow diagram.

Title readings and deletion of duplicates resulted in 137 articles that were shared between the authors for further consideration and bilateral agreement in terms of suitability for inclusion in the next review step. The articles’ abstracts were read by each author (MV, HS) independently and those articles with a possibility of discussion on ADRs in the Norwegian medicines management context were selected (*n* = 22). Their full texts were obtained from the Norwegian and UK libraries and underwent a careful assessment to select those with a precise focus on the study topic (*n* = 6). For example, articles with a focus on medication process, quality improvement, multidisciplinary interventions, and prescription and administration of drugs rather than ADRs reporting, monitoring, and handling were excluded. Also, the reference lists of the selected studies was searched manually, but no more articles were identified. Therefore, the number of articles was reduced to six for further evaluation and appraisal with respect to methodological transparency and soundness based on the Enhancing the QUAlity and Transparency of health Research (EQUATOR) tools [31]. The systematic review process is presented using the preferred reporting items for systematic reviews and meta-analysis (PRISMA) statement [32] (Figure 1). The characteristics of the articles selected for inclusion in this review are presented in Table 2. 

## 3. Results

### 3.1. General Description of the Studies

Of the selected studies, one was conducted in the Netherlands with the inclusion of data from Norway [33] and the remainder were undertaken in Norway [34,35,36,37,38]. Three articles [34,36,37] were in the Norwegian language and the rest [33,35,38] were published in English. They all used a cross-sectional descriptive design for data collection and analysis. The findings of the selected studies on ADR reporting and handling in the Norwegian healthcare context were presented under three central aspects of ‘patient reporting schema’ [33,36], ‘healthcare provider reporting schema’ [33,34,36,37], and ‘current pharmacovigilance system’ [33,35,36,37,38]. The characteristics of the studies’ findings were summarized in Table 3. 

### 3.2. Patient Reporting Schema 

The introduction of ADR reporting in Norway by patients was started relatively late in 2010. Only an electronic form was available, and this did not accept reports on unregistered products. This electronic reporting form was very sophisticated and was characterized by completeness checks, data importation directly to a database, presence of free-text and mandatory spaces in the form, and a drop-down list for the selection of co-medication. However, no drop-down lists to select ADRs were included and there was no opportunity for follow-up with patients regarding their ADR reports [33]. In the review and analysis of all ADR reports submitted to NoMA by patients from 2010 to 2013 (*n* = 755), female patients reported ADRs in 63% of cases and were most commonly in the age range of 20–29 years (29%) or 30–39 years (23%). Analgesics, including tramadol, codeine, diclofenac, and ibuprofen, were the most commonly mentioned drug group in the ADR reports. There were 1.1 psychotropic drugs per ADR report, and adverse mental and neurological reactions, such as dizziness, anxiety, depression, insomnia, and suicidal thoughts, were the most common suspected adverse reactions reported [36]. 

### 3.3. Healthcare Provider Reporting Schema

In regional centers in Norway only reports from healthcare professionals were assessed for causality, with personalized feedback provided [33]. In the review and analysis of all ADR reports submitted to NoMA by healthcare providers from 2010–2013 (*n* = 9629), 58% of healthcare personnel reports were for female patients, most frequently in the age group 0–9 years (17%) and 60–69 years (13%). The number of suspected drugs per ADR report was 1.4, and the most frequently mentioned drug group in the ADR reports was vaccines, where non-specific symptoms and local reactions were the typical suspected adverse reactions [36]. 291 reports were submitted to RELIS by healthcare providers from 2013–2015 (*n* = 409) on oral anticoagulants, including warfarin, dabigatran, rivaroxaban, and apixaban. Rivaroxaban had the highest number of reports (6.5 reports per 1000 anticoagulant users). The patients were mainly older than 70 years of age (76%); 91 patients experienced cerebral haemorrhages, blood clots, cognitive impairment, headache, and hair loss. The highest number of fatal outcomes were related to rivaroxaban (1.1 deaths/1000 users). Comorbidities, older age, and polypharmacy contributed to increased seriousness of side effects and patient death [37]. 

The study of ADR reporting by healthcare professionals on plant-based products including herbal dietary supplements and plant-based drugs e.g., Hypericum perforatum, Ginko biloba, and Valeriana, in the period 2003–2012 showed 260 submitted ADR reports to RELIS, of which 250 reports were related to herbal dietary supplements. There were 10 reports on plant-based drugs, all reported by pharmacists. Overall, doctors most frequently reported ADRs to the RELIS (*n*= 196, 75%). Severe ADRs were reported in 42% of cases including hypersensitivity (*n* = 71), hepatic damage (*n* = 56), and interactions with anticoagulant drugs (*n* = 20). Also, 72% and 20% of the reports specified to 53 years old women and patients with the age ≥70 years [34]. 

### 3.4. The Current Pharmacovigilance System

Norway shared only reports classified as serious with the EudraVigilance database. The electronic report was imported into the database, thereby saving the organization time and ensuring data completeness and facilitating assessment. Also, the financial burden of human resources influenced the decision by the Norwegian Medicines Agency on prioritization and time allocation for handling individual reports. No medical confirmation of the patient reports was made, but patients could contact the Norwegian Medicines Agency to actively follow-up their information. It was impossible to share patients’ reports with the primary healthcare provider [33]. The spontaneous reporting system by healthcare providers could not be used to compare the incidence of ADRs associated with different medicines within the same class [37]. ADR reports from patients were more varied than those from healthcare providers. Overall, spontaneous patient ADR reporting supplemented healthcare providers ADR reporting [36]. 

ADR reports from 2004–2013 from the EudraVigilance database (*n* = 14,028) were combined with dispensing data (*n* = 800 million) from the Norwegian Prescription Database. Dividing report numbers for each prescribed medicine with the number of users was used to calculate the medicine-specific consumption-adjusted ADR rates to identify medicines with higher risks of safety issues. A high number of ADR reports involved diclofenac, olanzapine, diazepam, warfarin, sildenafil, and methylphenidate. The increased ADR reports and the medicine-specific consumption-adjusted ADR rate for atorvastatin in 2010 reflected a true increase in the number of reports rather than the number of users: 27 different ADRs, with at least one instance, were reported. These included dizziness, nausea, diarrhoea, feeling abnormal, and anxiety in women and aggression, self-injurious behaviors, cerebral infarction, palpitations, attention deficit hyperactivity disorder, hypertension, and vomiting in men. Many of the most commonly used drugs had more than 500,000 users and less than 100 ADR reports; this was due to either underreporting or rarity of serious ADRs. 78% of all ADRs were reported within seven days [38]. 

The RELIS database, as the network of four regional medicine-information and pharmacovigilance centers where pharmacists asked questions and provided feedback to healthcare professionals regarding drug-related questions and ADRs, along with and the Norwegian ADR database (as a reference) were searched in the time period 2003–2012. Lyrica^(R)^ (pregabalin) and drug abuse after its marketing in Norway in September 2004 was selected to assess its safety-related reports. Accordingly, 5427 (26%) of 21,071 parallel questions and answers between pharmacists and healthcare providers in the RELIS database concerned ADRs, which were considered references in 4% of reports in the Norwegian ADRs database. On the other hand, the Norwegian ADRs database was used as a reference in 7% of parallel questions and answers regarding ADRs. Eleven questions and 13 ADR reports concerned Lyrica^(R)^ and different aspects of its abuse: reports described dose escalation problems, craving, and withdrawal reactions, which led to the detection of new drug-safety problems [35]. 

## 4. Discussion

This review has described the various methods used for reporting, monitoring, and handling ADRs in the Norwegian healthcare system. In general, emphasis was placed on the role of patients, healthcare providers, and innovative systems for ADRs reporting and monitoring. 

### 4.1. Patients vs. Healthcare Providers’ Reporting of ADRs

The study findings demonstrated how patients could be involved in ADR reporting and the barriers to patients’ participation in terms of their knowledge, active involvement and participation in medicines management, and feedback on reports. Rather, there was an emphasis on the extent of healthcare providers’ collaboration with the reporting of ADRs along with the use of their knowledge and expertise for the active monitoring of ADRs and medicines adverse side effects. Since low reporting rates of ADRs contribute to major delays in the identification of medicines management issues [18], patients’ reports have potential to improve the comprehensiveness of data collection by healthcare providers, as needed for any pharmacovigilance system. Comparisons between reports by patients and those by healthcare providers mostly highlight different points of view that can enrich spontaneous reporting data [39,40,41]. Variations in reporting processes and inclusion criteria for schemes and report types are responsible for differences between patients’ and healthcare providers’ reports [42]. Patients often reported more suspected ADRs to more medicines than did healthcare professionals, who often focused on patient-related information such as weight and height. Healthcare professionals usually reported the more serious reactions that lead to hospitalization, life threatening conditions, or death [43,44,45].

It has been reported that patients may not be aware of ADR reporting systems and may be confused about how and where to report. While patients are encouraged to report known ADRs to prevent similar suffering in other patients, they need to increase their familiarity with reporting processes to improve the quality and quantity of reporting [46]. To improve patients’ attitudes toward the reporting of ADRs, there is a need to increase public awareness campaigns to address the importance of reporting. By providing feedback to patients with regard to their reporting, as well as involving patients in decision making based on the results of reports, patient awareness should be increased [47]. One strategy could be to design a single official reporting system for both patients and healthcare providers, and measures should be taken to improve the interfacing of patients’ and healthcare providers’ reports to handle the potential increase in the number of reports [48]. In addition, all healthcare providers, including nurses who spend the most time with patients and collect most data on patients’ bedside conditions, should be educated on how to participate in reporting ADRs as only multiple reports can improve the impact of the pharmacovigilance system [3,4,20,49,50].

### 4.2. The Need for ADR Profiles for Identifying, Documenting and Reporting ADRs

The study findings showed the use of innovative methods and data collection methods for providing a comprehensive picture of ADRs in the Norwegian healthcare context. Some barriers to the effective implementation of the ADR reporting and monitoring system could be financial constraints, absence of clinical confirmation of patients’ reports, impossibility of sharing patients’ reports with primary healthcare providers, and inability to compare reports from different sources. The development and implementation of protocols for ADR monitoring as an efficient intervention to improve medicines management can contribute to lower reporting costs. Mechanisms for reporting and recording ADRs should not only provide information about the total number of reports, but also their severity, unexpectedness, and the degree of causality attributed to ADRs [51]. Variabilities in data fields used to report ADRs hinder the comparability of collected data from different reporting systems. Therefore, a common standardized dataset characterized by quality, comparability, and reporting rates can optimize drug safety surveillance efforts [52]. Moreover, regulation at the system level needs the inclusion of comprehensive, systematic, and regular patient checking for undesirable adverse effects of medicines. In this respect, the Adverse Drug Reaction (ADRe) profile, led by nurses, with the involvement of the multidisciplinary team, provides a suitable tool to achieve improved patient care and increased understanding of the impact of ADRs [3]. Modern electronic health records systems can assist healthcare professionals with completing ADR reporting. They are characterized by being easily accessible on the web and offer the possibility of sending emails or including direct hyperlinks to healthcare professionals’ desktops [18]. For instance, spontaneous ADR reporting is a pharmacovigilance method in Canada supervised by the Canadian Vigilance Program at Health Canada. ADR reports submitted to the system are sent by post, telephone, or via the internet, and are used to detect medication safety alerts [53]. Such systems also enable collecting ADRs reports from patients at their own homes, which is particularly important for those who live in remote areas, to allow direct contact with healthcare providers based in urban areas [17,54].

## 5. Conclusions

The results of this systematic review have described initiatives for ADR reporting, monitoring, and handling in the Norwegian healthcare system. The results emphasize the need for data collection systems to provide a comprehensive picture of ADRs. ADR reporting could be improved by: facilitating patients’ participation in reporting ADRs, and sending them feedback on their reports; sharing reports with primary healthcare providers, to enhance healthcare providers’ active collaboration with ADR reporting; implementing existing ADRe profiles for interactive reporting between patients, nurses and healthcare providers [3,4]; assessing reports in terms of quality and impact on the patient and the healthcare system, and facilitating access to reporting systems in inpatient and outpatient settings. More studies also are needed to find how ADRe pharmacovigilance profiles can improve the safety of medicines management in Norway.

## Figures and Tables

**Table 1:** The search strategy and results of different phases of the study (2010–2019).

Databases	Total in Each Database	Title Selection	Abstract Selection	Full-Text Appraisal
PubMed [including Medline]	5703	18	2	2
Scopus	85	15	1	1
Embase	27	5	0	0
Cinahl	196	0	0	0
Web of Science	250	7	1	0
Cochrane	46	3	0	0
Norat	68	26	8	3
Idunn	81	4	0	0
SweMed	103	59	10	0
*Manual search/backtracking references*	-	-	-	0
Total	6559	137	22	6

**Table 2:** List of studies selected for data analysis and synthesis in this systematic review.

Title	Authors	Year	Country	Aim	Methods
Experiences with adverse drug reaction reporting by patients: an 11-country survey.	van Hunsel et al. [33]	2012	Netherlands with the inclusion of data from Norway	To review the methods of patient reporting of adverse drug reactions (ADRs) in 11 countries and to compare different aspects of their experiences aimed at describing current practice.	A survey based on telephone interviews, e-mail discussions, and field visits.
Bivirkninger av plantebaserte produkter	Nergård [34]	2013	Norway	To describe the reports of ADRs of plant-based products in Norway from 2003–2012.	A retrospective cross-sectional study of reported ADRs from plant-based products in Norway 2003–2012.
Joint medicine-information and pharmacovigilance services could improve detection and communication about drug-safety problems	Schjøtt & Bergman [35]	2014	Norway	To describe the potential of the RELIS’s dual service to improve detection and communication of drug-safety problems.	Searching the RELIS database for question-answer pairs about ADRs using the Norwegian ADRs database.
Patient reporting of adverse drug reactions in Norway 2010–2013	Fjermeros et al. [36]	2015	Norway	To review patients’ reports of ADRs to the Norwegian Medicines Agency since 1 March 2010.	A cross-sectional retrospective study of ADRs reporting of patients to the Norwegian Medicines Agency (NoMA).
Bivirkninger ved bruk av antikoagulasjonsmidler i 2013-15 (Anticoagulant-associated adverse drug reactions in 2013–2015)	Eek et al. [37]	2018	Norway	To obtain a better insight into the ADRs profiles of the new direct-acting oral anticoagulants (DOACs).	A retrospective cross-sectional study of registry data (RELIS database of ADRs and the Norwegian Prescription Database (NorPD)).
Adverse drug reaction reporting: how can drug consumption information add to analyses using spontaneous reports?	Svendsen et al. [38]	2018	Norway	To combine ADRs reports with drug consumption data to demonstrate the additional information.	Combining all Norwegian ADR reports 2004–2013 from the EudraVigilance database (*n* = 14.028) with dispensing data from the Norwegian Prescription Database (more than 800 million dispensed prescriptions during 2004–2013).

**Table 3:** Characteristics of the studies’ findings on Adverse Drug Reactions (ADRs) reporting and monitoring.

Studies/Characteristics	Number of Reports	Size of Population Covered	Type of Reporting	Age of Patients	Gender of Patients	Most Commonly Reported Events	Patient Reporting	Healthcare Provider Reporting	Pharmacovigilance System
van Hunsel et al. [33]	30 consumer reports per month (14% of total)	No data	Electronic	No data	No data	Only on registered drugs	Sophisticated system without follow up	Causality assessment and personal feedback	Reporting only serious events
Nergård [34]	260 reports associated with plant-based products from 2003–2012	No data	No data	Average age of 52 years; 20% of the sample was >70 years	72% female	Hypersensitivity reactions (27%), hepatic events (20%), and interactions with anticoagulants (8%)	Not relevant, only reports from healthcare providers included	No data	RELIS database
Schjøtt & Bergman [35]	5427 (26%) of 21,071 question-answer pairs, and 791 (4%) of a total of 22,090 reports in the Norwegian ADR database	No data	Electronic	No data	No data	Dose escalation, craving, and withdrawal reactions to Lyrica^®^ (pregabalin)	No data	Provision of feedback by pharmacists and clinical pharmacologists to healthcare professionals	Norwegian ADR database and the RELIS database
Fjermeros et al. [36]	755 reports from patients and 9629 reports from healthcare staff in the time period of March 2010–December 2013	No data	No data	Average age not given. The patients most commonly reporting were in the age range of 20–29 years (29%), whereas healthcare providers’ reports most frequently concerned those aged 0–9 years (17%)	63% female among the patient reports, 58% female among the healthcare staff reports	Adverse mental and neurological reactions were commonly reported by patients, while healthcare providers reported mostly on general symptoms and local reactions.	No data	No data	Norwegian ADR database (NoMA)
Eek et al. [37]	409 reports on ADRs associated with anticoagulants in the time period of June 2013–May 2015	Approximately 145,000 anti-coagulant users	No data	Average age was 75–80 years	44% female	Cerebral haemorrhage, haemorrhage in skin/muscle/joint/mucous membranes and gastrointestinal haemorrhage	Not relevant, only reports from healthcare providers included	No data	Norwegian ADRs database (NoMA) and RELIS database
Svendsen et al. [38]	14.028 from the EudraVigilance database and 800 million from the Norwegian Prescription Database	Data from 5.1 million different persons.	Electronic	No data	22,351 female and 39,391 male users of methylphenidate	Physical and psychological symptoms in both males and females	No data	No data	EudraVigilance database, Norwegian prescription database

ADR: Adverse drug reaction.
